# Using Social Media to Maximize the Research Impact of Surgeons: Exploratory Linguistic Analysis

**DOI:** 10.2196/68004

**Published:** 2026-06-05

**Authors:** Samuel Berding, Emilie Green, Ania I Rynarzewska, Shane S Robinson, Nelson A Royall, Terence Jackson

**Affiliations:** 1 Graduate Medical Education, Northeast Georgia Medical Center, 743 Spring Street, Gainesville, GA, 60501, United States, 1 678 983 5533; 2 Biology Department, Texas Christian University, Fortworth, TX, United States; 3 Georgia College & State University, Milledgeville, GA, United States

**Keywords:** social media research, linguistic cues, research impact, surgical research, scholarly communication

## Abstract

**Background:**

Surgeons work in a progressive field where communicating research is vital to advancing health care and enabling meaningful interactions among clinicians. It also contributes to societal impact, increases access to information, and reduces misinformation. Additionally, there can be barriers to accessing papers. Social media enhances research impact through sharing scholarly work and improving its translation into clinical practice, but little is known about how to design specific posts to maximize research impact through language.

**Objective:**

The purpose of this study was to determine the linguistic cues that optimize research impact among surgeons through Twitter (subsequently rebranded X). Additionally, this research combines the linguistic features of the posts and article access to determine their unique contributions.

**Methods:**

An exploratory linguistic analysis of 84 posts extracted from Twitter was conducted, which shared scholarly activity by 17 of the most-followed surgeons. The linguistic cues were measured on a continuous scale, computed from the percentage of each linguistic cue used in the text, and reported as mean (SD). Regression analysis and analysis of covariance were conducted to determine which cues influenced research impact and to estimate the potential association with study accessibility (open vs restricted access).

**Results:**

Analyzed tweets were highly analytic (mean 94.77, SD 9.00), moderate in clout (mean 42.69, SD 19.84), low in tone (mean 20.06, SD 33.91), suggesting negative tone use, and low in authenticity (mean 19.52, SD 24.50). Results suggest that a high use of formal language negatively impacts readership and citations. Analytical language was indirectly associated with readership (β=−0.296, 95% CI −423.57 to −59.95; *P*=.01) and citations (β=−0.524, 95% CI −0.442 to −0.187; *P*<.001). Linguistic clout had a positive association with readership (β=0.260, 95% CI 8.58-186.91; *P*=.03), and tone in tweets had a negative association with readership (β=−0.317, 95% CI −138.52 to −5.39; *P*=.04). Negative language tone was found to increase the impact of research. With respect to linguistic cues and study accessibility, the results also suggest that the number of citations was impacted by readership (*F*_1,66_=4.11, 95% CI 2.459E-06 to 0.003; *P*=.047) and analytic linguistic cues (*F*_1,66_=18.77, 95% CI −0.402 to −0.149; *P*<.001) used in the post, but the association of open (mean 3.04, SE 1.062) versus restricted access (mean 1.83, SE 0.716) was not statistically significant (*F*_1,66_=0.877, 95% CI 0.405-3.266; *P*=.352).

**Conclusions:**

This research is the first to explore article accessibility and linguistic cues used in creating posts that share research on social media to determine their influence on research impact, making this study both innovative and unique relative to existing studies in the surgery field. Through language, the medical field can expand its impact and encourage dialogue between scientists and the public, thereby increasing scientific and societal contributions while reducing the negative effects of limited article access.

## Introduction

### Background

Social media presents numerous opportunities and threats to medical practitioners, researchers, journals, health care institutions, and society [1]. Health care and medical influencers, content creators, and even governmental health care messaging are often not grounded in science and likely arise from a misunderstanding of research findings. This contributes to misinformation, misconceptions, and the miseducation of patients and the general public, which are already prevalent on social media [2]. The frequency of social media use can amplify these issues [3]. The dissemination of medical and health-related information on social media poses risks to individuals and societies, as it is often mixed with misinformation found across various platforms [4].

Social media misinformation is defined as a combination of factual information blurred with falsehoods [5] and can increase health care skepticism, avoidance, and decreased trust in health care [3]. Twitter (subsequently rebranded X) has the highest prevalence of medical misinformation, and medical treatments, including surgery, are underexplored. Only 7% of studies on medical treatments have been evaluated, as opposed to 32% of vaccine-related studies [6]. However, an analysis of surgery-related content on social media (YouTube specifically) found that about a third of the videos contained misinformation, and about half provided no patient education [7]. This highlights the need for the proliferation of a social media environment with reliable information and patient education. Despite these efforts, the field of surgery fails to offer recommendations for the professional use of social media to advance its practice [8].

The way misinformation is created and shared is often exacerbated by the lack of trust in health care [5], making the causes and effects of misinformation complex and nuanced. For those reasons, sharing medical research in an understandable way with the public is more important than ever. Medical professionals can play a role in correcting misinformation on social media, mitigating its potentially negative impact [9].

At the same time, social media provides a powerful opportunity for the medical community to share medical and health-related information, thereby improving societal impact and elevating practitioners’ professional status [1]. For example, medical professionals who are actively present on social media and share their wealth of knowledge can improve overall health-related information sharing, increase doctors’ involvement in underserved areas, mentor medical students, and remain committed to lifelong learning [10]. One area of social media sharing involves disseminating research and providing early access to information from medical conferences. This can improve societal access to information, potentially combat misinformation, and enable timely sharing with other clinicians and medical researchers.

The prevalence of misinformation on social media and its potential adverse impact creates a need for interventions to limit the further magnification of the impact of social media users’ exposure to health care misinformation [11]. Medical researchers call on public health and medical professionals to correct misinformation present on social media by proactively sharing credible and factual information [2]. Medical and health care–related information sharing can additionally elevate practitioners’ status [10]. Thus, in addition to providing the opportunity to disseminate information and increase access to health care, social media enables medical professionals to establish themselves as professionals and experts in the field, beyond that of holding a medical degree or position.

### Gaps in Research

While the importance of social media in medical fields and health care, particularly with respect to vaccine information and disease prevention [12], is well established, surgery as a field still lags in its engagement with social media. This gap needs to be remedied. In fact, it has long been argued that social media is a necessary component of surgical practice for the reasons mentioned above, including mentorship, collaboration, rapid information sharing, patient education, and provider-patient relationship building [7,13]. It is important that this gap is remedied by medical and health care practitioners specifically. A study on posterior cruciate ligament injury and subsequent surgery revealed that social media content shared by practitioners or other medical sources had higher *JAMA* (*Journal of the American Medical Association*) scores, particularly with respect to the Global Quality Score, compared to content shared by nonpractitioners [14]. *JAMA* and Global Quality Score were also lower for content shared regarding hip arthroscopic surgery and were classified as poor quality information [14].

### Importance of Research

Given the importance of exposure to quality research in fighting misinformation, increasing access for underserved communities to health-related information, as well as sharing current medical information with the community, medical students, and medical professionals, the way the impact of research is measured is somewhat outdated and misguided. Additionally, artificial intelligence (AI)-powered search engines tend to favor open-access research, contributing to barriers to information access and limiting the research impact of restricted-access journal studies [15]. Currently, research impact is measured only by citations and the number of publications, which focus on elevating the researchers’ status and measuring their contribution to the body of knowledge [16]. In this exploratory research, we argue that the medical field should focus more on reach than on citation alone, shifting the focus to expanding readership. An emerging area of evaluating research impact is altmetrics, which focuses on social media as a source of research metrics [17]. Twitter has been an evolving platform for the dissemination of medical research, with its citations, downloads, and reads tracked. Improvements in these metrics have been linked to promotion on social media [18]. We argue that while these metrics are used to measure the dissemination of research, the barrier of open-access versus closed-access studies limits the purpose of disseminating research to advancing our understanding. Thus, a study may be shared and spread through social media, but depending on its accessibility, it may not be readable by all who interact with the post.

Social media has increased its use, specifically in the field of surgery, to help enhance individual profiles by engaging with other surgeons through virtual discussions of research and surgical education using hashtags that help users target their discussions to a closed group [19]. As social media continues to emerge with new, advancing techniques, including robotic procedures, many physicians use these platforms as a source of professional development with others in their field [20,21]. These interactions help improve knowledge translation—the process of moving from research to implementation in medical practice—which is essential as surgical techniques and patient presentations continue to evolve and challenge the current level of knowledge [18,22]. Research additionally shared by tweets has shown a significant increase in citations, thus confirming its potential to improve the rate of translational knowledge [23].

When it comes to medical information dissemination on Twitter, 3 general types of tweets exist [24]. Grajales et al [24] noted that: (1) substantive tweets are independently understandable (eg, a tweet with an abridged title or author of a paper, a brief comment, and a link to the publication, or a headline teaser to a blog); (2) conversational tweets are fragments of a new or ongoing conversation that draw on professional or personal interests or comment on current events; and (3) hybrid tweets, which are substantive and conversational at the same time (eg, “discussing my supervisor’s newest *Nature* publication at the Mahoney and Sons pub”).

This research specifically focuses on dissemination in the surgery subfield because “surgeons should learn how to use novel communication technology to advance the field and further professional and public interaction” and to improve the quality of surgery-specific information shared on social media [25,26]. Currently, posts sharing research and education are made by individual accounts as well as accounts of the journals that publish the research to increase reach [27]. Recent analyses have shown that journals with Twitter accounts that promote studies incur better metrics and, thus, greater dissemination [28]. Furthermore, personal accounts promoting studies have a greater impact on metrics than the accounts of journals themselves [29]. Social media has proven to be an effective tool for disseminating research. Beyond its impact on the medical field and researchers’ status, scientific research ought to have an impact on society at large, including public health, policymakers, patients’ trust in health care, and knowledge dissemination beyond narrower medical specializations, many of which do not use specialized medical language [30]. Thus, research involving text analysis of language used in social media posts disseminating published research offers an opportunity to understand how the language cues used in those posts contribute to research impact on a far broader scale than previously measured. The findings go beyond whether to engage in research sharing on social media; rather, they provide specific suggestions for how to share research to increase its impact.

### Goal of Study

The study is particularly important because little is known about the impact of linguistic cues on key research metrics beyond access. More specifically, the current state of knowledge lacks an understanding of how the writing of individual social media posts can influence research impact in terms of citations and altmetrics, with each metric offering a unique benefit.

Ultimately, the purpose of this research is to share novel ideas and techniques so that they may be applied directly to benefit medical researchers and journals striving to increase the dissemination and impact of research, while also providing the media and the general public with easy access to breaking medical findings. Linguistic analysis may show that certain linguistic cues can influence post interactions, thereby affecting the extent of research dissemination and improving the rate of translational knowledge. Limited knowledge of how to use social media and linguistic cues to disseminate medical research restricts its use as a dissemination tool.

## Methods

### Participants

This retrospective study evaluated the posts of 20 surgeons and surgical societies identified by OncoAlert as the most influential tweeters on Twitter [31]. The most recent 20 tweets from these 20 surgeons and societies were filtered to include only those that contained scholarly research papers and were posted in English. To provide insights into the sources of text data, only publicly available information disclosed on the profiles was used to ensure subject anonymity. At the time of data collection, account location was not accessible on Twitter. Each account focused on surgery, predominantly but not limited to oncological surgery. A combination of verified and unverified accounts was used to reduce the “verification”-based effect. The size of each account’s following ranged from just under 1000 followers for smaller accounts to nearly 150,000 followers for larger accounts. Each post was then analyzed to identify tweets that met the inclusion criteria while excluding others (eg, unrelated posts or those not discussing research). Posts that did not include links to published research were excluded. The initial sample yielded 477 observations; after the initial extraction, only posts that shared research papers were retained. Given that scraping was conducted on existing social media posts within the limits of Twitter’s terms of service, a maximum of 20 tweets per account was extracted, and no missing data were recorded. Prior to the change in Twitter ownership, researchers were able to easily extract data from Twitter for research purposes. However, after the change in ownership, open access to Twitter was terminated. This resulted in a decline in data access and data transparency, reducing researchers’ ability to produce a large sample size for research purposes [32].

### Study Design

This retrospective, exploratory study evaluated tweets from 17 accounts that met the inclusion criterion of containing any tweets relevant to the study’s purpose, yielding 84 usable observations after excluding posts that did not meet the research criteria. This study uses content analysis with manual coding, and the sample size is considered appropriate. Content analysis can be both qualitative and quantitative. For qualitative content analysis, 1 to 30 cases are considered appropriate [33]. Based on the initial plan, 20 individual accounts and the most recent 20 posts from each were selected for analysis before undergoing manual coding, resulting in 17 accounts being analyzed. For Linguistic Inquiry and Word Count (LIWC) analysis, which quantifies qualitative text data, the requirements focus on the text size [31]. For this research, 2665 words were analyzed, with 84% of each text unit (tweet) containing 20 or more words, meeting the reliability criteria [34,35]. Additionally, 8% (for a total of 91%) of tweets had 19 words. Only 1 tweet had 11 words.

Based on the content analysis of posts, 59 (70.2%) posts included a visual element: a study preview page, an image, or an audiovisual abstract. Furthermore, 26 (30.9%) of the eligible posts were retweets from other accounts. Posts included links to a paper, with 70 of the 84 (83.3%) posts tagging another account. More specifically, tagged accounts included the journal in which the research was published and the coauthors. Thirty-four (40.47%) of the eligible posts included at least 1 hashtag. For each tweet relevant to the study, information regarding open access versus restricted access, number of reads, number of citations, reshares, and posts’ likes was additionally extracted from Twitter or manually collected from journals where the research was published. All tweets containing research papers were analyzed based on LIWC analysis, which extracted 117 linguistic variables, including 8 summary variables, followed by a statistical analysis using SPSS (version 29; IBM Corp). An α level of less than or equal to .05 was used for all statistical tests.

### Ethical Considerations

The study was submitted to and granted exemption by the institutional review board at Brenau University (2418606‐1). Due to the study’s retrospective nature, the institutional review board deemed that no consent forms were necessary, as the data collected were already publicly accessible before the study was conducted. Additionally, no compensation was provided for study participation. While the data are publicly accessible, the data reported in this paper do not specify any of the specific accounts reviewed for the study to ensure anonymity and compliance with research ethics. Therefore, the study data were deidentified from publicly available sources voluntarily shared on Twitter.

### Outcome Measures

Research impact was operationalized as the total number of citations and readership (reads) per study. Citations are often used as a measure of research impact [16], making them an appropriate measure for this purpose. For a consistent evaluation, each study mentioned in the 84 tweets was manually reviewed, and the total number of citations and study reads was recorded from the initial journal site that published the study. Thus, the dataset merges the language of social media posts with journal metrics.

### Method for Linguistic Analysis

Text data from Twitter was analyzed using LIWC, a validated software tool that quantifies text in terms of complexity, emotion, cognition, social processes, health cues, and leadership language, extracting about 80 variables from the text data [36]. While internal validation is not typically conducted due to the nature of text structure, the software has been previously validated across fields using surveys and experimental designs [37]. However, a manual validation was conducted by randomly selecting 10% of the dataset and manually calculating the percentage of key variables present in the extracted text, resulting in a 100% match between the manual and LIWC-calculated results. Additionally, the reliability of LIWC was assessed against the required criteria, as it is suggested that the majority of text used for analysis should be around 20 [35]. The reliability criterion was met as described in the “Study Design” section. Analytical thinking, clout, authenticity, and emotional tone were used in the analysis. Each of the individual variables that are later summarized into the variables of interest is first computed into scores that are measured in percentages ranging from 0 to 100 [38]. The individual items indicative of various psychological processes are then converted into summary scores, which are standardized composite variables transformed to a scale from 1 to 100 [37]. Within these parameters, scores considered low in these summary variables are less than 50, and those considered high are greater than 50. However, a tone below 50 indicates a negative tone, while a tone above 50 indicates a positive tone. Analytical thinking (analytic) captures the extent to which people use words that suggest a formal, logically thought-out pattern of thinking and is usually associated with academic success and academic dissemination [39]. Clout is defined as the way one shows confidence in their writing by sharing information related to the subject [40]. Authenticity refers to genuine language, with those who use it being perceived as true to self. This is an important measure because social media users have a preference for authenticity, which can be threatened by the desire for self-presentation [41]. Surgeons may seek to project a particular professional image on social media, which may inadvertently undermine their perceived authenticity. Finally, emotional tone refers to the individual’s use of emotional language in the text being shared and can range from negative to positive emotions. Perceived emotionality differs because the perceived emotionality measure assesses how one feels about the information being shared and, in this case, how supportive they are of the information they share with their followers [42]. These measures were used to best describe and quantify the language used in each tweet, along with word complexity measures, including word count (WC), words per sentence (WPS), and big words, to determine which cues and structures were most associated with reaching impact. ANOVA and ANCOVA tests were used to analyze the data. This type of analysis is considered appropriate for the analysis of LIWC data [34].

## Results

### Overview

The analysis focuses on the impact of summary variables that encompass multiple linguistic cues and language complexity in social media posts disseminating surgical research. These include analytic language (a metric of logical, formal thinking), clout (language of leadership and status), authentic language (language that induces perceived honesty and genuineness), tone (ranging from negative to positive), and determinants of text complexity, which include big words (words consisting of ≥7 letters), the average WPS, and the WC of each post. Descriptive statistics, including mean and SD, suggest very high analytic cues indicative of high formal and logical thinking (mean 94.77, SD 9.00). Authentic language was low, averaging in the lower quintile (mean 19.52, SD 24.50). The tone was also in the lower quintile (mean 20.06, SD 33.91), with values under 50 indicative of negative language. Finally, the language of leadership or confidence (clout) was just below the midpoint of the scale (mean 42.69, SD 19.84). Additional summary statistics and variables related to text length and complexity (WPS, WC, and big words) can be found in Table 1. In light of the findings described below, the high value placed on analytic language is problematic because of its negative association with impact. Clout language also offers opportunities for improvement. The opportunities and potential threats will be discussed in the Discussion section of this paper.

**Table 1.** Descriptive statistics associated with tweet characteristics by linguistic cues (N=84).

Linguistic cue	Min-max	Mean (SD)
Word count	11-57	31.73 (12.213)
Analytic	36.67-99	94.7725 (9.00360)
Clout	3.95-97.73	42.6876 (19.83876)
Authentic	99-99	19.5245 (24.49750)
Tone	99-99	20.0552 (33.91182)
Words per sentence	48-48	23.8949 (10.29585)
Big words	61.54-61.54	37.3788 (8.85206)

### Predicting Readership

Six summary measures were used as predictors to determine their impact on readership. To test the association of each summary variable with readership, we conducted a regression analysis. The model predicting readership from these key variables successfully predicted study readership (adjusted *R*^2^=0.11; *F*_7,67_=2.28; *P*=.04), where the adjusted *R*^2^ provides insights into the proportion of variance in the outcome (11%) explained by the model. Analytic linguistic cues (β=–0.296; B=−241.76, 95% CI −423.57 to −59.95; *P*=.01) and tone had an inverse association with readership (β=−0.317; B=−71.96, 95% CI −138.52 to −5.39; *P*=.04), with the standardized β coefficient providing information about the change in the outcome variable with a standardized unit increase in a predictor variable while holding other independent variables constant. In the case of analytic cues and emotional tone, the outcome (readership) declines with the increase in the prevalence of these cues. In contrast, clout had a positive association with readership (β=0.260; B=97.74, 95% CI 8.58-186.91; *P*=.03), as seen in Table 2. For scientific transparency, we also report unstandardized regression coefficients (unstandardized coefficients) and SE in Table 2, which provide information on the change in the outcome variable with each unit increase in an independent variable when other independent variables are held constant, and an estimate of the SD or uncertainty of the sample statistic. It is worth noting that while authentic language was not significant at an α level of .05 (*P*=.055), it is trending toward significance. Given the small sample size, the impact of authentic language should not be readily excluded.

**Table 2.** Predicting readership from linguistic cues used in Twitter (subsequently rebranded X) posts^a^.

Model	Unstandardized coefficients		Standardized β coefficients	*t* test (7,67)^b^	*P* value	95% CI for β
	B	SE				
(Constant)	31334.68	10875.13	—^c^	2.88	.01	9627.83 to 53041.53
Analytic	−241.76	91.09	−0.296	−2.65	.01	−423.57 to −59.95
Word count	−3.57	104.48	−0.006	−0.03	.97	−212.12 to 204.97
Clout	97.74	44.67	0.260	2.19	.03	8.58 to 186.91
Authentic	21.79	36.09	0.071	0.60	.55	−50.25 to 93.82
Tone	−71.96	33.35	−0.317	−2.16	.03	−138.52 to −5.39
Words per sentence	−126.78	103.18	−0.165	−1.23	.22	−332.72 to 79.16
Big words	−173.15	124.42	−0.199	−1.39	.17	−421.49 to 75.20

a Dependent variables (readers): *R*=438; *R*2=0.192; *F*_7, 67_=2.277; *P*=.04.

b Note: The *F* test (*df*=7,67) was used for overall significance, and the 2-tailed t test was used for individual coefficients (*df*=1,67).

c Not applicable.

### Predicting Citations

To accurately predict citations, the initial model incorporated readership due to its likely impact on citations and study accessibility, given the potential barrier due to the lack of accessibility. The initial analysis of all summary measures of linguistic cues, readership, and study accessibility resulted in a nonsignificant impact for several cues. The nonsignificant cues were removed, and a parsimonious model was retained. Statistical analysis based on regression analysis of linguistic cues on citations suggests an inverse association of analytic linguistic cues (β=–0.524; B=−0.315, 95% CI −0.442 to −0.187; *P*<.001) while retaining the remaining cues and controlling for all summary linguistic factors in the model (adjusted *R*^2^=0.259; *P*<.001). This association was substantive. Next, to determine the relationship between accessibility and citations, an analysis of covariance was conducted (*R*^2^=0.316; adjusted *R*^2^=0.285). The results suggest that the number of citations was affected by readership (*F*_1,66_=4.11, 95% CI 2.459×10^–6^ to 0.000; *P*=.047) and analytic linguistic cues (*F*_1,66_=18.77; B=−0.276, 95% CI −0.402 to −0.149; *P*<.001) used in the caption, but the association of open access (mean 3.04, SE 1.062) versus restricted access (mean 1.83, SE 0.716) was not statistically significant (*F*_1,66_=0.877; B=1.205, 95% CI −1.364 to 3.775; *P*=.352) when controlled for analytic language cues and readership, as seen in Table 3. Additionally, Table 3 also includes the intercept, which provides information about the value of the predicted variable when independent variables are set to zero, and partial η, which provides information about each independent variable’s effect size—the unique proportion of variance in a dependent variable that is associated with a specific independent variable.

**Table 3.** Impact of linguistic cues used in Twitter (subsequently rebranded X) posts, readership, and study accessibility on citations^a^.

	Mean square/B	*F* test (*df*)	*P* value^b^	Partial η^2^
Corrected model	249.022	10.179 (3, 66)	<.001	0.316
Intercept	523.958/27.52	21.416 (1, 66)	<.001	0.245
Reads	100.619/0.00015	4.113 (1, 66)	.047	0.059
Analytic	459.277/–0.275	18.772 (1, 66)	<.001	0.221
Study accessibility	21.466/1.205	0.877 (1, 66)	.35	0.013
Error	24.465^c^	—^d^	—	—

a *R*2=.316 (adjusted *R*2=0.285).

b Computed using α=.05.

c Error value is mean square only.

d Not applicable.

## Discussion

### Principal Findings

This exploratory study achieved its purpose by retrospectively evaluating posts shared on Twitter to identify the best linguistic cues for optimizing research impact among surgeons. The study found that study readership was positively associated with the language of leadership, known as clout, and inversely associated with analytical language and a more positive tone. These findings suggest that leadership language is most effective in increasing the overall readership of published work, ultimately maximizing public and professional consumption of scientific research. However, an increase in positive tone and analytical language may reduce readership, thereby reducing the overall consumption of scientific research.

Furthermore, this research combined the linguistic features of social media posts and the study’s access status to determine their unique contributions to study impact, using citations as a measure of impact. The findings suggest that the number of citations was positively associated with readership and inversely associated with analytic linguistic cues in the caption, but the association between open and restricted access was not statistically significant, underscoring the importance of linguistic cues while reducing the previously known associations of study access on impact. Previous research suggests that open access positively affects citations, but publishing open access often carries economic implications for researchers and their institutions [43]. This research suggests that the linguistic features of a social media post can overcome a study’s open- or restricted-access status, reducing the economic burden on researchers and their institutions to publish open access.

By accomplishing the goals set for this study, this research is the first to examine the associations between study accessibility and the linguistic cues used to create Twitter posts when sharing research papers on social media, as well as the relationship between these cues and research impact, measured by citations and readership. This exploratory study was conducted in the context of surgical research dissemination to address the existing gap in research specific to this subfield. More specifically, in this research, we estimated that formal language has a substantial negative impact on readership and citations in research publications. The negative impact of this type of language on both readership and citations suggests the need to use less formal language when sharing medical research on social media platforms such as Twitter. This is a counterintuitive finding that requires further explanation. Marketing literature suggests that the effectiveness of language used is context-specific, whereas formal language may be more appropriate when the matter discussed is serious [44]. At the same time, formal cues are linked to platform credibility, which tends to be the lowest on Twitter [45].

Contrary to the association with analytic language, readership is positively impacted by clout, which is the language of leadership and status. While clout is a summary variable, it can be difficult to understand what it means and how to use language to increase clout scores [46]. Scholars suggest that clout language is indicative of levels of confidence in writing [47]. The higher the score, the more confident the post is, whereas a lower score indicates greater doubt. Furthermore, prior studies have found that language associated with cognitive processing along with its subcategories, such as discrepancy or differentiation, is negatively associated with clout scores [46]. For these reasons, confident but not overly formal writing in social media posts sharing medical research should limit the use of discrepant statements, as they may introduce doubt. Thus, increasing the use of confident language will directly and indirectly positively impact readership and citation, respectively.

Finally, as the findings suggest, a more positive tone in social media posts reduces readership directly and citations indirectly. Since our tone variable ranges from negative to positive tonality, the findings indicate that using negative language positively impacts readership. This finding aligns with current research on the effects of negative language on social media, which suggests increased readership of studies that are incongruent with one’s opinion and negatively framed. Negative language also amplifies the dissemination of these studies on social media [48]. However, caution should be exercised when using a negative tone to increase social media engagement with published research. A well-documented tendency among social media users to pay attention to negative information on social media platforms is known as the negativity bias [49,50]. While this study’s findings align with this social media effect, participation in negative discourse may contribute to further polarization [51] and accidental spillover effects onto the published paper, potentially introducing negative associations. For these reasons, based on the findings of this research, it is recommended that researchers who intend to increase the impact of their research by engaging in social media sharing consider increasing the linguistic confidence of their posts by reducing post discrepancies and differentiation cues, which can introduce doubt rather than confidence. Additionally, it is recommended that researchers reduce their reliance on formal language. While research between open and restricted access suggests that open-access studies outperform restricted-access studies [37]—a trend further magnified by AI-powered search engines—this study found that linguistic cues had a statistically more significant impact on readership and citations than study accessibility.

Increasing the impact of medical research by sharing studies on social media can offer an avenue to increase research impact, as acknowledged in scientific fields [52] and also improve research readership and engagement in both scientific and public spheres [53]. It could also serve as a psychological inoculation against misinformation present on social media, increasing the ability to recognize misinformation and its spread [11]. Through the use of language to which the social media audience may be most receptive, researchers, journal outlets, and medical associations are likely to become catalysts for knowledge sharing, increase research impact, and encourage conversation not only among scientists but also in public spheres, allowing for increased scientific and societal impact [30]. At the same time, social media sharing can help reduce the gap in journal access that AI-powered search engines tend to magnify.

### Limitations

While this study offers a novel approach to using social media to maximize research impact, it is not without limitations. Our biggest limitation in this analysis is the small sample size of 84 observations; however, we wanted to analyze surgeons with the greatest social media success and influence to better understand which cues were most important. The source of this limitation is tightly linked to changes in Twitter operations that limit data extraction. Of the extracted data, not all posts qualified for the study and had to be excluded if they did not discuss published research. Despite the sample size limitation, the goal of this study was still accomplished, particularly given its exploratory nature. The second limitation of this study was that the observed tweets were shorter, with fewer words. Larger texts allow the analysis to uncover more cues that contribute to the findings. However, this limitation is strictly tied to how social media operates, as individuals may be more inclined to read shorter texts. Nonetheless, short social media texts, like those on Twitter, were successfully used in contributing to the understanding of study-relevant outcomes [54]. Thus, social media posts capture attention despite their brevity, and social media research on the content of posts and comments has considered them an appropriate source of data and information. Furthermore, while text size might be viewed as a limitation, it is the state of social media platforms that is outside of the authors’ Twitter that continues to limit the size of textual posts, although subscription options allow for more text characters and longer posts.

### Conclusions

Our exploratory findings suggest that the use of language should be considered when disseminating academic publications through social media platforms, regardless of whether the information is open access. While high clout was associated with readership, negative language contributed to a relationship that led to more citations and increased research impact. Although readership does not automatically lead to citations, it statistically influences citations; readership is also important to optimize, as it helps improve the knowledge and evidence-based practice of both experienced and newly trained practitioners [55]. While these findings apply to surgical fields, future research should expand to identify optimal linguistic cues for other medical specialties and determine whether they observe similar associations. Future research might also consider comparing the linguistic cues used by social media influencers and medical professionals to determine any differences in language and impact between the groups.

## Abbreviations

AI: artificial intelligence
*JAMA*: *Journal of the American Medical Association*
LIWC: Linguistic Inquiry and Word Count
WC: word count
WPS: words per sentence
